# Signal Transduction across the Nuclear Envelope: Role of the LINC Complex in Bidirectional Signaling

**DOI:** 10.3390/cells8020124

**Published:** 2019-02-04

**Authors:** Miki Hieda

**Affiliations:** Department of Medical Technology, Ehime Prefectural University of Health Sciences, 543 Takooda, Tobecho, Ehime 791-2102, Japan; mikihieda@gmail.com; Tel.: +81-89-958-2111; Fax: +81-89-958-2177

**Keywords:** LINC complex, nuclear envelope, mechanotransduction, SUN protein, KASH protein

## Abstract

The primary functions of the nuclear envelope are to isolate the nucleoplasm and its contents from the cytoplasm as well as maintain the spatial and structural integrity of the nucleus. The nuclear envelope also plays a role in the transfer of various molecules and signals to and from the nucleus. To reach the nucleus, an extracellular signal must be transmitted across three biological membranes: the plasma membrane, as well as the inner and outer nuclear membranes. While signal transduction across the plasma membrane is well characterized, signal transduction across the nuclear envelope, which is essential for cellular functions such as transcriptional regulation and cell cycle progression, remains poorly understood. As a physical entity, the nuclear envelope, which contains more than 100 proteins, functions as a binding scaffold for both the cytoskeleton and the nucleoskeleton, and acts in mechanotransduction by relaying extracellular signals to the nucleus. Recent results show that the Linker of Nucleoskeleton and Cytoskeleton (LINC) complex, which is a conserved molecular bridge that spans the nuclear envelope and connects the nucleoskeleton and cytoskeleton, is also capable of transmitting information bidirectionally between the nucleus and the cytoplasm. This short review discusses bidirectional signal transduction across the nuclear envelope, with a particular focus on mechanotransduction.

## 1. Introduction

**Figure 1:**
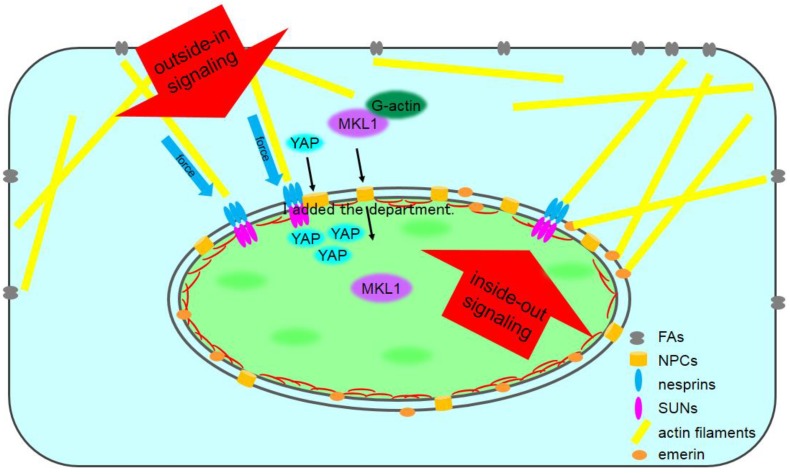
A model of Linker of Nucleoskeleton and Cytoskeleton (LINC) complex-mediated inside-out and outside-in signaling pathways. An enormous number of signaling pathways are integrated within cells. Outside-in signaling pathways, which originate within the cytoplasm or extracellular environment and terminate within the nucleus, can be roughly categorized into two groups. In the first pathway, mechanical stimuli are directly transferred to the nucleus via the LINC complex. The second pathway utilizes transcription factors such as Yes-associated protein (YAP) and megakaryoblastic leukemia 1 (MKL1) that shuttle between the cytoplasm and the nucleus through the nuclear pore complex (NPC). More importantly, the nucleus also plays a role as an information submission source. Inside-out signaling results in the transfer of signals from the nucleoplasm to the cytoplasm across the nuclear envelope (NE). SUN domain: Sad1/UNC-84.

The cytoskeleton physically connects the plasma membrane with the nucleus; therefore, the nucleus responds to mechanical stimuli originating from outside of the cell after its propagation though the cytoskeleton. Integrins are receptors for extracellular matrix proteins and assemble into large macromolecular complexes known as focal adhesions (FAs), which connect the extracellular matrix to intracellular actin bundles [1]. An association between integrins, the cytoskeleton, and the nucleus was reported more than 20 years ago [2]. Integrins also act as mechanosensors in the plasma membrane and are critical for the organization of multiple nuclear components, such as chromatin and nucleoli [3,4,5]. Investigation of the interconnected integrin–cytoskeleton–nucleus mechanotransduction pathway has largely focused on the outside-in signals that initiate at the plasma membrane and are transferred to the nucleus (Figure 1, inside-out signaling). However, recent data show that the nucleus also plays a role as an information submission source (Figure 1, inside-out signaling), which is critical for the cells to sense and respond to the mechanical properties of their environment [6,7,8].

The nucleus is mechanically integrated with the cytoskeleton via the Linker of Nucleoskeleton and Cytoskeleton (LINC) complexes, which are evolutionarily conserved nuclear envelope (NE)-spanning molecular bridges. The LINC complexes are comprised of the Sad1/UNC-84 (SUN) domain-containing proteins located on the inner nuclear membrane and the Klarischt/ANC-1/SYNE homology (KASH) domain-containing proteins, known as nuclear envelope spectrin repeat protein (nesprins), which are found on the outer nuclear membrane. Mammals encode five SUN proteins (SUN1~5) and six KASH proteins (nesprins-1–4, KASH5, and lymphocyte-restricted membrane protein) [9,10,11,12,13,14,15]. SUN proteins interact with lamins and chromatin in the nucleus, whereas nesprins associate with the various elements of the cytoskeleton in the cytoplasm [16,17,18]. Thus, the LINC complex has diverse functions, including nuclear migration [19], maintenance of the proper nuclear morphology and positioning of the nucleus [20,21], maintenance of the centrosome–nucleus connection via direct or indirect interaction [22,23,24,25,26], DNA repair [27,28], cell migration [29,30,31], and movement of chromosomes within the nucleus during meiosis [32]. In addition, the LINC complex transmits various signals from the cell surface to the nucleus [33] as well as from the nucleus to the cytoplasm [6,7,8]. This review summarizes the current understanding of bidirectional signal transduction across the NE, with an emphasis on the LINC complex and mechanical signaling from extracellular environment via FAs.

## 2. Outside-in Signaling across the NE

Outside-in signaling pathways across the NE, that originate within the cytoplasm or extracellular environment and terminate within the nucleus, can be roughly categorized into two groups. In the first pathway, mechanical stimuli are directly transferred to the nucleus via the LINC complex. The second pathway utilizes biochemical molecules that shuttle between the cytoplasm and the nucleus through the nuclear pore complex (NPC). In this section, these two pathways which mediate with mechanotransduction from the extracellular environment and the cytoplasm to the nucleus will be discussed. Lamins are intermediate filament proteins and form the nuclear lamina scaffold, which localizes adjacent to the inner nuclear membrane. Lamin A and lamin C (Lamin A/C) participates in various cellular responses to mechanical stimuli, including regulation of transcription, modulation of nuclear and cellular stiffness, and regulation of nuclear morphology [34]. Because this review focuses on the transducers of the LINC complex, for information about functions of lamin A/C in mechanotransduction, please refer to an excellent review that has been recently published by Osmanagic-Myers et al. [35].

### 2.1. Responses of the Nucleus Induced by Mechanical Stimuli

When mechanical force is applied to the extracellular domain of integrins present on the plasma membrane, alterations of the nuclear morphology are induced, indicating that mechanical stress can be transmitted from the extracellular matrix (ECM) to the nucleus [2]. Other mechanical stimuli, such as stretch and compression, impact nuclear shape and the organization of nucleoplasmic structures, such as chromatin and nucleoli [36,37,38,39,40,41]. Here, actin filaments and intermediate filaments are involved in the transfer of mechanical stimuli to the nucleus. Poh et al. showed that when a force of several nanonewtons was applied to cell surface integrins using magnetic beads coated with Arg-Gly-Asp (RGD) peptide, which is an established integrin ligand, it induced rapid (less than 1 s) dissociation of two major structural proteins, coilin and SMN, from the Cajal body [41]. Disruption of the actin cytoskeleton or depletion of lamin A/C abolishes this response, suggesting the presence of an integral signaling pathway between integrins and nuclear structures [41]. The application of mechanical stress to the integrins within the plasma membrane triggers an increase in cellular stiffness [42,43]. A similar stiffening response was recently demonstrated by the Burridge group to occur in isolated HeLa cell nuclei that were exposed to mechanical forces applied via magnetic tweezers that pulled on magnetic beads coated with anti-nesprin-1 antibodies, which were bound to the outer nuclear membrane [44].

In addition to being able to sense and respond to externally applied mechanical stimuli, cells themselves exert mechanical forces upon their environment via the transmission of actomyosin-generated tension through cell–cell and cell–substrate adhesions. Recently, two research groups showed that the actin filament-severing proteins actin depolymerizing factor (ADF) and cofilin were essential for normal nuclear structure in tissues and in cultured cells. ADF and cofilin-1, which are products of separate genes, negatively regulate non-muscle myosin-II activity through competitive inhibition for binding to F-actin [45,46]. Depletion of both ADF and cofilin-1 causes various nuclear defects including aberrant nuclear morphology, discontinuities in the nuclear lamina, and a reduction of heterochromatin at the nuclear periphery (detected by the accumulation of H3K27me3, H4K20me3 and topo3). Depletion of nesprin-2 giant (nesprin-2G) or lamin A/C using small interfering RNA prevents nuclear abnormalities from occurring in cells simultaneously depleted of cofilin-1 and ADF showing that cytoplasmic actomyosin-generated mechanical stimuli are transferred to the nucleus and regulate nuclear morphology.

In addition to its impact on nuclear architecture, mechanical stress influences the posttranslational modification of histones (e.g., acetylation and methylation) as well as the dynamics and intranuclear localization of heterochromatin [47]. The genome is exposed to mechanical stresses originating from the cytoplasm, which can be transmitted either via the LINC complex or through the contact sites between the NE and chromatin. These stresses affect the localization of histone-modifying enzymes that indirectly modulate chromatin organization via epigenetic modifications. For example, inhibition of actomyosin contractility induces the nuclear translocation of the histone deacetylase HDAC3, resulting in a decrease in the levels of histone acetylation [48,49]. Another example of this phenomenon is that T-cell adhesion through integrin α4/β1 induces the recruitment of the histone methyltransferase G9a to the NE, resulting in an increase in the level of the histone post-translational modification H3K9me2/3 [50]. These altered histone modifications can contribute to physical properties of the nucleus. For instance, nuclear peripheral heterochromatin may enhance the structural robustness of the nucleus and strengthen its ability to resist physical stress, such as mechanical forces exerted during cell migration or in the mechanically active tissues [51,52]. Moreover, altered higher order chromatin organization induced by changes in histone modification has a significant effect on gene expression [53] and thus mechanical stress can also regulate gene expression [54]. For information related to transcriptional regulation by mechanical stimuli, please refer to recent excellent review written by Uhler and Shivashankar [55].

### 2.2. Outside-In Signaling via the LINC Complex

Mechanical stretch induces the proliferation of C2C12 myoblast cells, but the disruption of the LINC complex by overexpression of dominant-negative nesprin or SUN proteins constructs suppresses this phenomenon [56]. This result indicates that there is a mechanotransduction pathway to the nucleus through the LINC complex. The perinuclear actin cap covers the apical surface of the nucleus and regulates nuclear shape in adherent cells [36]. Nesprin-2G and nesprin-3 anchor the perinuclear actin cap, which is a highly organized array of parallel contractile actin filament bundles that contain phosphorylated non-muscle myosin II regulatory light chain and the F-actin crosslinker α-actinin, to the apical surface and lateral sides of the interphase nucleus [57]. The perinuclear actin cap is not equivalent to the dorsal perinuclear actin cables because of their highly specified components [36]. Wirtz’s group demonstrated that overexpression of a dominant negative KASH construct or the depletion of the LINC complex proteins nesprin-2G or nesprin-3 inhibits the assembly of the perinuclear actin cap and that perinuclear actin cap associated focal adhesions (ACAFAs) differ from conventional FAs in morphology, size, and spatial distribution [33,36,58,59]. Intriguingly, the perinuclear actin cap is formed in response to shear stresses 50 orders of magnitude lower and faster than biochemical stimulation in adherent cells [33]. These data clearly show the existence of an interconnected physical pathway of the integrin–cytoskeleton–LINC complex, which enables ultrafast mechanotransduction from the extracellular environment to the nucleus. In addition, transmembrane actin-associated nuclear (TAN) lines are linear arrays of nesprin–2G/SUN2 LINC complexes, which associate with perinuclear actin cables on the dorsal surface of nuclei in migrating fibroblasts and myoblasts. Formin homology 2 domain containing 1 (FHOD1), Samp-1, and torsinA are TAN line components [60,61,62,63]. TAN lines are required for rearward nuclear movement during centrosome orientation in migrating fibroblasts and myoblasts [29,60]. Moreover, Burridge’s group directly applied mechanical stimulus to nesprin-1 on the surface of isolated nucleus and demonstrated the existence of a mechano-transduction pathway into the nucleus via the LINC complex [44]. This study also revealed that lamin A/C and its binding partner, an inner nuclear membrane protein, emerin, participates in local nuclear stiffening, which is induced by mechanical stimuli. In response to the mechanical pulling of nesprin-1, emerin undergoes tyrosine phosphorylation by Src. This phosphorylation strengthens the connection between lamin A/C and the LINC complex, which is important for the expression of mechanosensitive genes and the assembly of stress fibers [44]. Using fluorescence resonance energy transfer-based tension biosensors, Arsenovic et al., (2016) demonstrated that nesprin-2G is subject to mechanical tension in adherent fibroblasts with the highest level of force on the apical and equatorial planes of the nucleus. This tension was observed to be reduced in fibroblasts obtained from patients with Hutchinson–Gilford progeria syndrome [64].

### 2.3. Outside-In Signaling through the NPC

Mechanical stress applied to the cell surface induces alterations in protein conformation and post-translational modification as we as the assembly of protein complexes. All these processes can modulate protein localization and activate biochemical signaling pathways [65,66]. Megakaryoblastic leukemia 1 (MKL1), which is also known as myocardin-related transcription factor A (MRTF-A) or MAL, is a mechanosensitive transcriptional coactivator of the serum response factor (SRF) [67]. Its localization is regulated via changes in actin polymerization. MKL1 is localized in the cytoplasm by its interaction with monomeric actin and by its constitutive nuclear export [68,69,70,71,72,73]. Mechanical stimulation induces RhoA-mediated actin polymerization and the dissociation of MKL1 from monomeric actin, resulting in the accumulation of MKL1 in the nucleus and the up-regulation of SRF-inducible genes including actin, SRF, and vinculin [68,69,70,71,72,73]. These proteins regulate cellular motility and contractility [74]. In addition, emerin is a crucial modulator of actin polymerization and absence of emerin or its binding partner lamin A/C results in disturbed actin dynamics and impaired MKL1 signaling [75,76]. Moreover, nuclear actin was observed to be more mobile in *LMNA*^−/−^ cells than in wild-type controls. The nuclear translocation and downstream signaling of MKL1 is impaired in cells lacking in lamin A/C, but not in the LINC complex [76]. This is consistent with the suggestion that lamin A/C, but not the LINC complex, is required for the activation of the mechanosensitive genes which encode the proteins vinclulin, tailin, and Egr-1 in heart tissue and cultured fibroblasts from mice [37,77,78].

Recent exciting work demonstrates that mechanical stimuli directly regulate nuclear-cytoplasmic transport via the NPC [79]. The inner lumen of NPC comprises a disorganized, flexible meshwork of proteins containing phenylalanine–glycine repeats (FG-Nups), which suppresses protein diffusion [80,81,82]. Yes-associated protein (YAP) is a mechanosensitive transcriptional regulator that has physiological and pathological functions [83,84,85]. The intracellular localization of YAP is regulated by the Hippo signaling pathway [86] and mechanical stresses such as ECM rigidity and shear stress [87,88,89,90,91]. Mechanical stress induces the nuclear translocation of YAP in a tailin-, actomyosin-, and LINC complex-dependent, but Hippo signaling-independent manner. Elosegui-Artola et al. demonstrated that there is a faster nuclear entry of YAP in cells grown on stiffer substrates. Nuclei in cells grown on stiff substrates are more flat than those in cells grown on soft substrates, and mechanical stimuli increase NPC permeability, allowing YAP to more readily enter the nucleus [79]. They also showed that the LINC complex was required for the increase in the nuclear translocation of YAP observed in cells grown on stiff substrates. It remains unclear which SUN protein(s) is involved in the regulation of NPC permeability, while SUN1 but not SUN2 colocalizes with NPC and interacts with components of NPC [31,92,93,94]. This mechanism might be generally applicable beyond YAP and further investigation of the interplay between outside-in signaling through the NPC and outside-in signaling via the LINC complex is needed.

## 3. Effects of NE on the Cytosolic Cytoskeleton

Recent evidence suggests that the LINC complex signals not only from the cytoplasm into the nucleus but also from the nucleus to the cytoplasm. In this section, effects of the LINC complex on the cytoskeleton and inside-out signaling through the LINC complex are discussed.

### 3.1. The LINC Complex Affects the Organization of the Cytoskeleton

Disruption of the LINC complex impairs the dynamics and organization of the actin cytoskeleton and consequently cellular mechanics [29,36,37,58,77,95,96]. For example, expression of dominant negative nesprin alters the localization of actin and vimentin filaments as well as suppresses the migration of mouse embryonic fibroblasts [37]. The LINC complex-associated proteins, emerin, torsinA, and lamim A/C, also influence cytoskeleton dynamics. Expression of phosphoresistant emerin mutant decreases the number of actin bundles [44], while impaired lamin A/C function alters actin cytoskeleton around the nucleus when cells are cultured on a rigid substrate [77]. In addition, outer nuclear membrane-localized emerin associates with non-muscle myosin IIB and organizes actin flow for nuclear movement and centrosome orientation in migrating fibroblasts, suggesting a novel function for the nuclear envelope in organizing directional actin flow and cytoplasmic polarity [97]. TorsinA-depleted NIH3T3 fibroblasts displayed impaired retrograde flow of perinuclear actin cables [63]. Dysfunction of the LINC complex affects not only cytoskeletal organization, but also cellular adhesion [30,98,99]. Depletion of nesprin-1 in endothelial cells increases the number of FAs, traction forces and, nuclear height [30]. SUN2 contributes to the mechanical integrity of intercellular adhesions in mammalian epidermal keratinocytes [98]. Knockdown of the LINC complex components nesprin-2 or SUN1 leads to a substantial increase in the prominence of the adhesion domain at the opposite end of the invadopodia [99]. Moreover, LINC complexes influence microtubule (MT) organization as well. Gomes group has used the proximity-dependent biotin identification (BioID) method and revealed that muscle cell specific nesprin-1 isoform, nesprin-1α interacts with several centrosomal proteins including Akap450, Pcm1, and pericentrin. They also showed that nesprin-1α regulates MT nucleation [100]. In *Drosophila*, KASH domain protein Klarsicht regulates MT stability and integrin localization during collective cell migration [101]. These results indicate a physical role for the nucleus as anchoring point for cytoplasmic cytoskeletal elements.

### 3.2. Inside-Out Signaling via the LINC Complex

Recent papers have described several pathways in which the LINC complex signals to the cytoplasm, though detailed molecular mechanisms have not been well understood. Carroll’s group showed that SUN2 promotes the assembly of FAs by activating RhoA, while SUN1 antagonizes SUN2 and suppresses RhoA activation and FA assembly [6]. Coirault and her colleagues demonstrated that muscle precursor cells expressing mutant lamin A/C (*LMNA*^ΔK32^) or nesprin-1^ΔKASH^ had reduced ability to adapt to the rigidity of their environment. These cells exhibited contractile stress fiber accumulation, increased number of FAs and higher traction force on soft subtrates, which mimic physiological muscle stiffness. Inhibition of rho-associated protein kinase (ROCK) or a ROCK-dependent actin remodeling regulator, FHOD1, rescued the morphology of mutant cells, showing that functional integrity of lamin A/C and nesprin-1 is required to modulate activity of FHOD1 [7]. Chavrier and his colleges have shown that the nucleus-centrosome linkage of nesprin-2 and dynein adaptor Lis1 regulates the trafficking of MT1–MMP, one of matrix metalloproteinase family members, from the late endosomal/lysosomal storage compartments to the cell surface. The finding suggests that the LINC complex may also contribute to the persistence of malignancies and metastasis [8]. However, it has been shown that most of the LINC complex components are down-regulated in several types of cancer tissues ([102], unpublished data); thus, for the LINC complex to function during cancer metastasis, a more intricate regulation might exist. Emerin is present at both inner and outer nuclear membranes where it interacts with lamins and the cytosleketon, respectively [103]. The Wickström group recently showed that mechanical forces drive the enrichment of emerin in the outer nuclear membrane of epidermal stem cells, which is accompanied by the recruitment of muscle myosin IIA (NMIIA) to and the polymerization of actin at the nuclear surface. As a result, there is a reduction in the level of actin within the nucleus, resulting in the attenuation of transcription. They also show that the corresponding decrease of emerin at the inner nuclear membrane leads to a switch from H3K9me2/3 on constitutive heterochromatin to H3K27me3 as well as impaired anchorage of heterochromatin to the nuclear periphery [104]. Therefore, the NE may function as an integrator of cytoskeletal and nuclear functions.

## 4. Concluding Remarks

Recent data suggest that, like the cell–ECM boundary, the NE is a dynamic stimuli-sensitive interface between the cytoplasm and chromatin [105]. The LINC complex, which is localized at the NE, acts in a variety of signaling pathways between the cytoplasm and the nucleus. Currently the mechanisms responsible for regulating the assembly of functional LINC complexes remain poorly defined, although several lines of evidence identify several candidates for LINC complex regulators including intraluminal calcium, the redox environment of the NE, torsinA, and ubiquitinylation [63,106,107,108]. Finally, an analogy between FAs and LINC complexes can be drawn. Similar to the mechanical stimuli-dependent assembly of FAs, mechanical stresses applied to the nucleus via the cytoskeleton recruit the LINC complex and lamin A/C to specific indentation sites under stress fibers that exert force on the NE [109]. In addition, heterochromatin-related histone modifications are interestingly upregulated prior to and during cell migration [52,110,111]. Thus, in a similar manner to integrin-mediated inside-out signaling, it can be hypothesized that the chromatin signature might regulate the functional assembly of LINC complexes and their ability to transduce signals across the NE.

## Abbreviations

NE	nuclear envelope
LINC complex	Linker of Nucleoskeleton and Cytoskeleton
lamin A/C	Lamin A and lamin C
MT	microtubule
SUN domain	Sad1/UNC-84
KASH	Klarischt/ANC-1/SYNE
nesprin	nuclear envelope spectrin repeat protein
MT	microtubule
FHOD1	formin homology 2 domain containing 1
FA	focal adhesion
NPC	nuclear pore complex
ADF	depolymerizing factor
Nesprin-2G	nesprin-2 giant
